# DUOX2 As a Potential Prognostic Marker which Promotes Cell Motility and Proliferation in Pancreatic Cancer

**DOI:** 10.1155/2021/6530298

**Published:** 2021-03-02

**Authors:** Meng Cao, Peng-Bo Zhang, Peng-Fei Wu, Qun Chen, Wan-Li Ge, Guo-Dong Shi, Jie Yin, Bao-Bao Cai, Shou-Ji Cao, Yi Miao, Kui-Rong Jiang

**Affiliations:** ^1^Pancreas Center, The First Affiliated Hospital of Nanjing Medical University, Nanjing 210029, China; ^2^Department of General Surgery, Nanjing Drum Tower Hospital, The Affiliated Hospital of Nanjing University Medical School, Nanjing 210008, China; ^3^Department of General Surgery, The Affiliated Hospital of Xuzhou Medical University, Xuzhou 221002, China; ^4^Pancreas Institute, Nanjing Medical University, Nanjing 210029, China

## Abstract

DUOX2 has been reported to highly express in several types of cancers. However, the prognostic significance and the biological function of DUOX2 expression with pancreatic cancer (PC) still remain unclear. The present study is aimed at investigating whether DUOX2 could act as a novel biomarker of prognosis and evaluating its effect on PC cell progression. The mRNA and protein expression of DUOX2 in PC cells and tissues were assessed by quantitative real-time PCR (RT-qPCR) and immunohistochemistry. The effect of DUOX2 expression on PC cell motility and proliferation was evaluated *in vitro*. The correlation between DUOX2 mRNA expression and clinicopathological features and its prognostic significance were analyzed according to the Gene Expression Profiling Interactive Analysis (GEPIA) website based on The Cancer Genome Atlas (TCGA) and the GTEx databases combined with our clinical information. According to bioinformatics analysis, we forecasted the upstream transcription factors (TFs) and microRNA (miRNA) regulatory mechanism of DUOX2 in PC. The expression of DUOX2 at transcriptional and protein level was dramatically increased in PC specimens when compared to adjacent nontumor specimens. Functionally, DUOX2 knockdown inhibited cell motility and proliferation activities. Our clinical data revealed that the patients had better postoperative overall survival (OS) with lower expression of DUOX2, which is consistent with GEPIA data. Multivariate analysis revealed that high DUOX2 expression was considered as an independent prognostic indicator for OS (*P* = 0.031). Based on Cistrome database, the top 5 TFs of each positively and negatively association with DUOX2 were predicted. hsa-miR-5193 and hsa-miR-1343-3p targeting DUOX2 were forecasted from TargetScan, miRDB, and DIANA-TarBase databases, which were negatively correlated with OS (*P* = 0.043 and *P* = 0.0088, respectively) and DUOX2 expression (*P* = 0.0093 and *P* = 0.0032, respectively) in PC from TCGA data. These findings suggest that DUOX2 acts as a promising predictive biomarker and an oncogene in PC, which could be a therapeutic target for PC.

## 1. Introduction

Pancreatic cancer (PC) has become one of the most lethal malignant diseases in the recent years. According to the latest global estimates, there are more than 331000 deaths due to PC per year, which accounts for 4.0% of all deaths [1, 2]. The main reasons for high mortality rate were low early diagnostic rate, low precautions, low metastasis detection, and poor response rate to radiotherapy and chemotherapy [3, 4]. But patients with early stage PC show no obvious clinical symptoms, and when once detected, the survival rate reaches to 78% if the pancreatic tumors were smaller than 2 cm [5]. Patients who have undergone surgical resection are prone to recurrence, and their prognosis remained very poor, strongly demanding new treatments [6, 7]. More importantly, little is known on the molecular pathogenesis and therapeutic targets of PC. Therefore, it is imperative to explore new molecular markers to prolong the survival time of PC patients.

Niacinamide adenine dinucleotide phosphate (NADPH) oxidases (NOX) are classified as the main family of catalytically active enzymes that belonged to a multisubunit protein complex with complex seven members, including NOX1-5 and dual oxidase (DUOX) 1 and 2 [8]. As a potent oncogene, the expression of DUOX2 has been shown to be significantly upregulated in several cancer types including lung, cervical, colon, rectal, stomach, and pancreatic cancers when compared with adjacent nonmalignant tissues [9–15]. DUOX2 could be upregulated mainly with the help of some inflammatory mediators in human tumors, causing accumulation of H_2_O_2_ and DNA damage through STAT6 and NF-*κ*B signaling [16–18]. In addition, DUOX2 induced the cascade of reactive oxygen species (ROS) cascade, activating the proinflammatory cytokine IFN-*γ* in human PC cells [19]. However, the role of DUOX2 in PC cell proliferation and other hallmarks have rarely been reported and require clarification. Therefore, we delved into the role of DUOX2 in PC progression.

Hence, in the present study, we aimed to investigate whether DUOX2 was involved in PC progression. In addition, the association of DUOX2 expression with patients' prognostic information by obtaining from the public databases and our clinical data analysis was evaluated. These results provide insights into a new diagnostic and prognostic biomarker and a therapeutic target for PC patients.

## 2. Material and Methods

### 2.1. Patients and Tissue Samples

A total of 86 pancreatic tumor tissue samples and corresponding adjacent normal tissue samples were collected from the Pancreas Center, the First Affiliated Hospital of Nanjing Medical University, China, from May 2016 to July 2017. Informed consent was obtained from all patients, and the protocol was approved by ethics committee of our hospital. 86 patients, including 62 males and 24 females, were followed up regularly until April 12, 2019, and the median follow-up time was about 23 months. Tumor stage was classified and relied on the American Joint Committee on Cancer (AJCC) TNM staging system, 7th edition [20]. Moreover, a majority of patients were mainly in stages I and II. The OS time was calculated from the time of operation till the last follow-up visit or death.

### 2.2. Bioinformatics Analysis

GEPIA is a time-saving and intuition web application that is used for gene expression analysis based on abundant data from TCGA and the GTEx databases (http://gepia.cancer-pku.cn/) [21]. DUOX2 mRNA expression in PC tissues and normal pancreatic samples was obtained by using GEPIA. In addition, the survival analysis with DUOX2 subgroup and the relationship between DUOX2 and clinicopathological information were also analyzed. In addition, Cistrome is a beneficial platform that contained large amounts of cis-acting binding information of transregulatory elements [22]. With the aid of Cistrome, 1557 transcription factors (TFs) were downloaded. By analyzing the correlation coefficient between the expression of DUOX2 and TFs and obtaining its corresponding *P* value, the top 5 of each positively or negatively regulated TFs (*P* < 0.01 and Cor > 0.2) were screened as potential regulatory TFs of DUOX2 in PC. A correlation coefficient value was classified as follows: strong correlation (Cor > 0.5), moderate correlation (0.5 > Cor > 0.3), and weak correlation (Cor < 0.3) [23]. Three online databases including two miRNA prediction databases: TargetScan 7.2 (http://www.targetscan.org/vert_72) and miRDB v6.0 (http://mirdb.org.) [24] and one miRNA validated database DIANA-TarBase v8 (http://www.microrna.gr/tarbase) [25] were utilized for predicting the potential regulatory miRNAs of DUOX2 in PC which presented simultaneously in these databases.

### 2.3. Cell Culture

Five human pancreatic ductal adenocarcinoma (PDAC) cell lines (PANC-1, BxPC-3, AsPC-1, CFPAC-1, and MIA-PaCa-2) and a normal human pancreatic ductal cell line (hTERT-HPNE) were purchased from the Shanghai Cell Bank (Shanghai, China). According to the recommendation of ATCC, both BxPC-3 and AsPC-1 cell lines were cultured in RPMI­1640 Medium (Life Technologies; Carlsbad, CA, USA) supplemented with 10% fetal bovine serum (FBS) (Life Technologies; Carlsbad, CA, USA) and 1% penicillin-streptomycin (HyClone, USA). However, PANC-1, CFPAC-1, and MIA-PaCa-2 were grown in Dulbecco's modified Eagle's medium (DMEM) (Life Technologies; Carlsbad, CA, USA) containing 10% FBS and 1% penicillin-streptomycin (HyClone, USA). In addition, the hTERT-HPNE cell line was maintained in DMEM, supplemented with 5% FBS, 10 ng/ml human recombinant epidermal growth factor (EGF), 5.5 mMD glucose (1 g/L), and 750 ng/ml puromycin. All cell lines were routinely cultured in a humidified incubator with 5% CO_2_ at 37°C.

### 2.4. Transient Transfection with Small-Interfering RNAs (siRNAs)

Before transfection, BxPC-3 and PANC-1 cells were seeded in 6-well plates. When the cell density reaches to ~60% confluency, siRNAs that are specific to DUOX2 were transfected using Lipofectamine™ 2000 (Invitrogen, Carlsbad, CA, USA) according to the manufacturer's instructions. Cells were transfected by two different specific siRNAs (siRNA Nos. 1406 and 2848, one nontargeting control siRNA (Gene Pharma, Shanghai, China)). After 48 h, the cells were collected from each well, and the transfection efficiency was detected by quantitative reverse transcription PCR (RT-qPCR) and Western blotting. The siRNA sequences are listed in Table 1.

### 2.5. RNA Extraction and Quantitative Real-Time PCR (RT-qPCR)

Total RNA was isolated from tissues and PC cell lines using Trizol reagent (Invitrogen, Carlsbad, USA). PrimeScript™ RT reagent kit (TaKaRa, Japan, RR036A) was used for reverse transcription in order to obtain a fast and efficient cDNA template synthesis following the manufacturer's instructions. After that, fast start Universal SYBR Green Master (Rox) (Roche, USA, 491385001) and an ABI stem plus system (Applied Biosystems) were utilized for RT-qPCR. Relative quantification of mRNA expression was performed by comparative Ct (2^−*ΔΔ*Ct^) method and normalized to ACTB. The results of RT-qPCR were independently performed thrice. DUOX2 and ACTB primer sequences are listed in Table 1.

### 2.6. Western Blotting Analysis

The cells in different groups were lysed in lysis buffer supplemented with protease and phosphatase inhibitors (USA, B14001 and B15001) and underwent centrifugation. Protein concentrations were determined using a BCA protein assay kit (Bio-Rad, Hercules, CA, USA), and equal amounts of protein were analyzed by SDS-PAGE on 8% gels. The gels were transferred onto a polyvinylidene difluoride (PVDF) membrane (Millipore Corporation, Billerica, MA, USA). After blocking for 2 h using 5% skimmed milk, the membranes were incubated at 4°C overnight with primary antibodies DUOX2 (Santacruz, CA, USA, sc-398681; 1 : 200 dilution) and GAPDH (Santacruz, CA, USA, sc-25778; 1 : 1000 dilution). Following washing with TBST, the membranes were incubated with HRP-conjugated secondary antibodies (1 : 10000 dilution) at 37°C for 2 h and then analyzed. Western blotting results were independently performed thrice.

### 2.7. Immunohistochemical (IHC) Staining

To verify the expression of DUOX2 in the PC tissues, 40 pancreatic tumor tissues and paired normal tissue specimens were obtained for IHC staining. All tissues were incubated with 3% H_2_O_2_ and sodium citrate buffer (pH 6.0) for 5 min and 20 min, respectively. Finally, the samples were incubated overnight at 4°C with DUOX2 polyantibody (biorbyt, UK, Orb41406), followed by incubation with secondary antibodies. The immunohistochemically stained sections were analyzed by two well-trained pathologists who were blinded to clinical information. The sections were then photographed under three random fields through a microscope.

### 2.8. Cell Counting Kit-8 Assay

PANC-1 and BxPC-3 cells at a density of 2 × 10^3^ cells/well were transfected with DUOX2-si1406 and DUOX2-si2848 before seeding into 96-well plates. At the same time on each day, 10 *μ*L of CCK-8 reagent (Dojindo, Kumamoto, Japan) supplemented with 100 *μ*L of nonserum medium was supplemented into each well and incubated for another 1 h at 37°C. Subsequently, each well was measured at a wave length of 450 nm. Each group had six duplicate wells and was repeated three times.

### 2.9. Transwell Migration Assay and Invasion Assay

The invasion and migration of pancreatic cancer cells were tested by using 8 *μ*m transwell chambers (Millipore Corporation, Burlington, MA, USA). For invasion assay, the upper chambers were coated with 1 : 8 diluted Matrigel and incubated at 37°C for at least 1 h. PANC-1 (4 × 10^4^) and BxPC-3 cells (4 × 10^4^) in different groups were starved in 200 *μ*L serum-free medium in the upper chamber with (invasion) or without (migration) Matrigel (Corning, No. 356234). The lower chambers were filled with 600 *μ*L culture medium containing 10% FBS, which act as chemoattractant. After incubation at 37°C for 24 h even 48 h, the cells were stained with 4% paraformaldehyde and 0.5% crystal violet solution for 30 min. Finally, the number of cells that migrated or invaded w counted in five selected areas under 100x magnification using an inverted microscope. Each result was repeated three times.

### 2.10. Statistical Analysis

All statistical analyses and graph presentation were performed using four kinds of software including SPSS version 25, Stata version 16, R version 3.6.3 (http://www.r-project.org/), and GraphPad Prism version 7.0. The quantitative data were presented as means ± SD. Comparison between two groups was performed using an independent Student's *t*-test (two-tailed) and chi-square test (*χ*^2^ test). The survival curves of PC patients were calculated using Kaplan-Meier method, and the differences in OS between high and low DUOX2 expression groups were calculated using logrank test. Univariate and multivariate analyses of these variables were performed using Cox proportional hazard regression model to calculate the relative risk among all the affecting factors. A two-sided *P* value of <0.05 was considered to be statistically significant.

## 3. Results

### 3.1. DUOX2 Expression Was Upregulated in PC Cells

**Figure 1:**
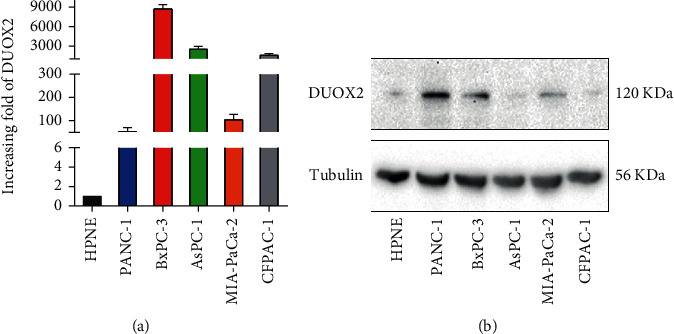
DUOX2 expression in five PC cells and normal pancreatic duct cell. (a) RT-qPCR was performed to analyze DUOX2 mRNA expression. (b) Western blot was used to detect DUOX2 protein expression. Data were presented as mean ± SD.

To compare the expression level of DUOX2 between 5 human pancreatic ductal adenocarcinoma (PDAC) cell lines (PANC-1, BxPC-3, AsPC-1, CFPAC-1, and MIA-PaCa-2) and a normal human pancreatic ductal cell line (hTERT-HPNE), RT-qPCR and Western blotting were performed. The results revealed that DUOX2 mRNA and protein showed an obvious upregulated expression in PANC-1 and BxPC-3 cell lines when compared to hTERT-HPNE cell line (Figures 1(a) and 1(b)).

### 3.2. DUOX2 Expression Was Upregulated in PC Tissues

**Figure 2:**
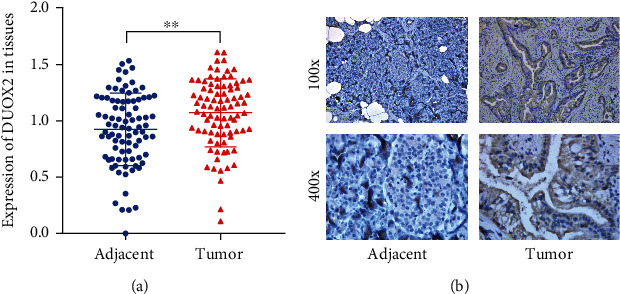
Upregulation of DUOX2 expression in PC tissues. (a) The mRNA expression level of DUOX2 was detected in tumor samples and adjacent normal tissues. (b) Representative photographs at 100x and 400x magnifications in tumor samples and paired adjacent normal tissues. Data were presented as mean ± SD. ^∗∗^*P* < 0.01.

To investigate the role of DUOX2 in pancreatic cancer progression, DUOX2 mRNA expression levels were examined in 86 PC tissues and paired normal adjacent tissues by RT-qPCR. The results showed that the transcriptional levels of DUOX2 expression were significantly increased in cancer tissues when compared to matched normal tissues (Figure 2(a)). To further analyze whether DUOX2 plays a role in human PC tissues, the protein expression level of it was analyzed by immunohistochemistry between 40 PC tissues and their corresponding normal tissues which were chosen at random. The results demonstrated that DUOX2 that is located in the cytoplasm showed an elevated expression in the tumor tissues when compared with adjacent normal samples (Figure 2(b)).

### 3.3. DUOX2 Expression and Its Correlation with Clinicopathological Features Obtained from the GEPIA Website in PC

**Figure 3:**
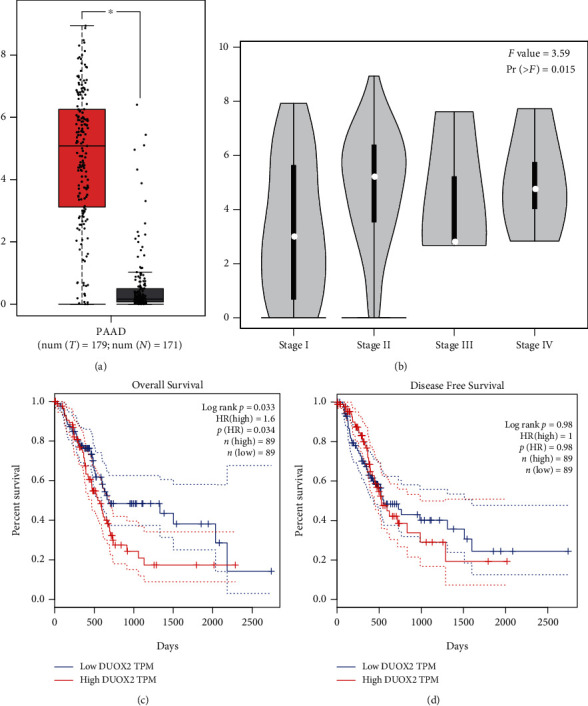
DUOX2 expression in PDAC and its association with clinicopathological features in the GEPIA website (data from TCGA and GTEx databases). (a) DUOX2 mRNA expression in GEPIA in tumor tissues and normal tissues. (b) The expression level of DUOX2 in all stages presented in violin pictures. (c) K-M survival curve showing the association of DUOX2 with overall survival. (d) K-M survival curve showing the association of DUOX2 with disease free survival. Data were presented as mean ± SD. ^∗^*P* < 0.05.

To investigate the expression of DUOX2 in PDAC and its relevance in PDAC patients' survival, we analyzed DUOX2 using the GEPIA website. We found that DUOX2 was significantly elevated in PDAC tissues compared with normal pancreatic tissues (Figure 3(a)). In addition, the expression of DUOX2 in PDAC in stage II according to AJCC was statistically different than in stage I (Figure 3(b)). Furthermore, we found that high DUOX2 expression in PDAC predicted shorter overall survival (OS) compared with low DUOX2 expression and showed significant difference (*P* < 0.05) (Figure 3(c)), whilst its expression correlated with disease free survival (DFS) was no significant difference (*P* > 0.05) (Figure 3(d)).

### 3.4. Relationship between DUOX2 mRNA Expression in PC and Clinicopathological Characteristics

**Figure 4:**
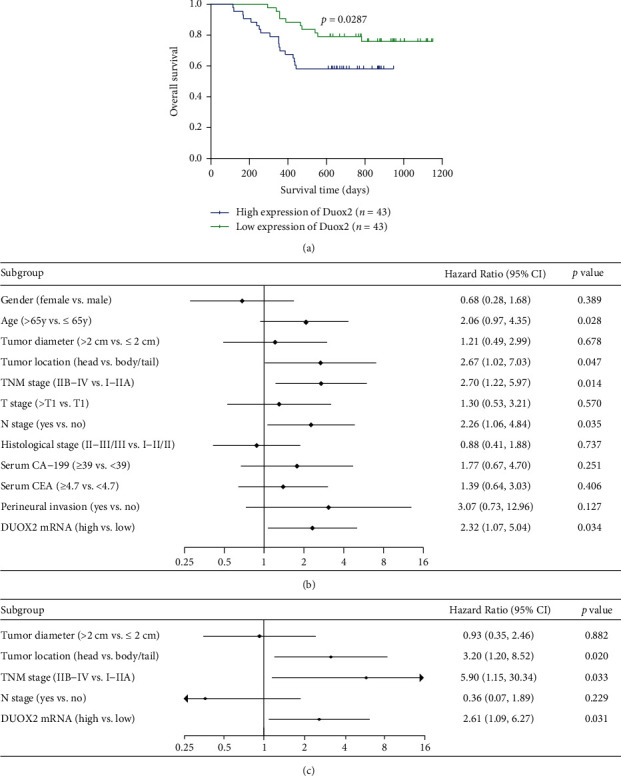
DUOX2 expression association with OS and Cox analysis clinicopathological features based on our data. (a) K-M survival curve revealing the expression of DUOX2 associated with overall survival. (b) Risk factors and (c) independent risk factors for overall survival in univariate and multivariate Cox regression analysis. Data were presented as mean ± SD; ^∗^*P* < 0.05.

To understand the inherent mechanism of DUOX2 abnormal expression in PC, 86 patients were enrolled to assess the prognostic significance of DUOX2. Among them, 58 were alive, and the remaining 28 patients were dead during the last follow-up visit. A threshold of DUOX2 median mRNA value was used to divide all patients into two groups: the high and low DUOX2 expression. Kaplan-Meier survival curves revealed that the patients with low DUOX2 expression (<1.122) had higher postoperative survival rate when compared to those with high DUOX2 expression (Figure 4(a), *P* = 0.0287, logrank test), suggesting the effects of DUOX2 on the prognostic performance of PC.

Next, the relationship between clinical pathological features and DUOX2 expression in patients with PC was also evaluated. As illustrated in Table 2, DUOX2 expression showed significant association with tumor diameter (*P* = 0.019) and TNM stage (*P* = 0.005), especially T and N stages (*P* = 0.041 and *P* = 0.001, respectively). These results indicated that low DUOX2 expression acts as a good prognostic predictor in patients with PC.

Cox regression analysis includes the following parameters: gender, age, tumor diameter, tumor location, TNM stage, T stage, N stage, histological stage, serum CA-199, serum CEA, perineural invasion, and DUOX2 mRNA. Univariable Cox regression analysis showed that DUOX2 expression (hazard ratio (HR) 2.318, 95% confidence interval (CI) 1.067–5.035, and *P* = 0.034), TNM stage (HR 2.697, 95% CI 1.219–5.968, and *P* = 0.014), N stage (HR 2.263, 95% CI 1.059–4.837, and *P* = 0.001), and tumor location (HR 2.671, 95% CI 1.015–7.031, and *P* = 0.047) were risk factors for OS, whereas multivariable Cox regression analysis revealed that high DUOX2 expression (HR 2.614, 95% CI 1.090–6.270, and *P* = 0.031), advanced TNM stages (HR 5.897, 95% CI 1.154–30.143, and *P* = 0.033), and head of pancreatic cancer (HR 3.201, 95% CI 1.202–8.518, and *P* = 0.020) were independent factors correlated with OS (Figures 4(b) and 4(c)).

### 3.5. Knockdown of DUOX2 Inhibits PC Cell Motility and Proliferation

**Figure 5:**
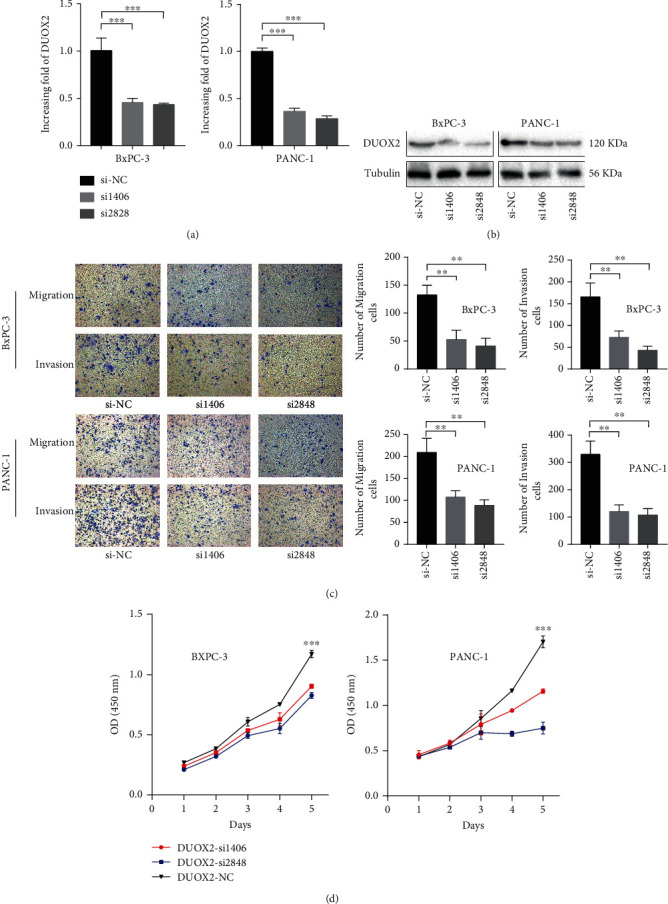
Knockdown of DUOX2 expression in PC cells (PANC-1 and BxPC-3) suppresses cell motility and proliferation. The expression of DUOX2 at (a) mRNA and (b) protein level with siRNA of DUOX2 interference. PANC-1 and BxPC-3 cell migration, (c) invasion, and (d) proliferation with siRNA of DUOX2 interference. Data were presented as mean ± SD. ^∗∗^*P* < 0.01 and ^∗∗∗^*P* < 0.001.

To investigate whether DUOX2 could regulate the function of PC cells, DUOX2 specific siRNAs were transfected into PANC-1 and BxPC-3 cells to silence the expression of DUOX2. As shown in Figures 5(a) and 5(b), Western blotting and RT-qPCR have confirmed the reduction of DUOX2 protein and mRNA expression in PANC-1 and BxPC-3 cells by knocking down it. Transwell assay was also performed to evaluate the effects of DUOX2 on the invasion and migration of PC cells. The results showed that low expression of DUOX2 inhibited PANC-1 and BxPC-3 cell migration and invasion (Figure 5(c)), suggesting the involvement of it in the progression of PC. Functionally, CCK-8 assay was carried out to evaluate cell proliferation between DUOX2-silencing cells and normal control cells. As shown in Figure 5(d), the results showed that the growth rate of PANC-1 and BxPC-3 cells was significantly attenuated after downregulating the expression of DUOX2 when compared with normal control cells.

### 3.6. Establishment of TF Regulatory Network

**Figure 6:**
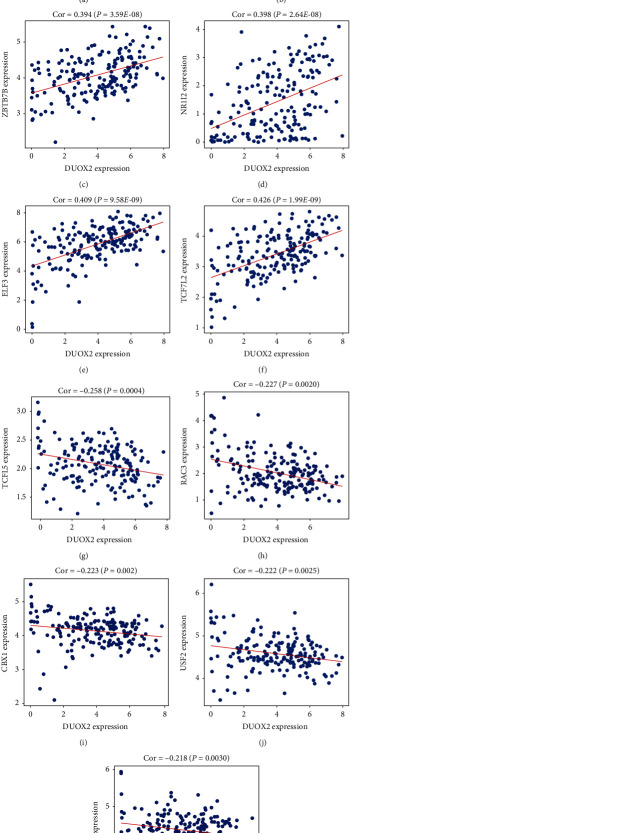
Prediction of TFs and DUOX2. (a) Each top 5 TFs positively and negatively regulating DUOX2. (b) ONECUT2, (c) ZBTB7B, (d) NR1I2, (e) ELF3, (f) TCF7L2, (g) TCFL5, (h) RAC3, (i) CBX1, (j) USF2, and (k) RPA2 correlation with DUOX2 mRNA expression. Data were presented as mean ± SD. ^∗∗^*P* < 0.01 and ^∗∗∗^*P* < 0.001.

To reveal the potential upstream regulatory mechanism of DUOX2 in PC, we calculated the connection between TFs and DUOX2 expression. Among 1547 TFs obtained from Cistrome database, the top 5 of each positively or negatively regulatory TFs satisfying the criterion (*P* < 0.01 and Cor > 0.2) were chosen as the potential TFs of DUOX2. As shown in Figure 6(a), transcriptional regulatory network of DUOX2 was built based on 10 TFs. Among them, ONECUT2, ZBTB7B, NR1I2, ELF3, and TCF7L2 were moderately and positively connected with DUOX2, whereas TCFL5, RAC3, CBX1, USF2, and RPA2 were weakly and negatively related to it (Figures 6(a)–6(k)). Furthermore, only USF2 expression was significantly associated with OS in PC according to the GEPIA database (data not shown).

### 3.7. Upregulation of hsa-miR-5193 and hsa-miR-1343 Lead to DUOX2 Downregulation

**Figure 7:**
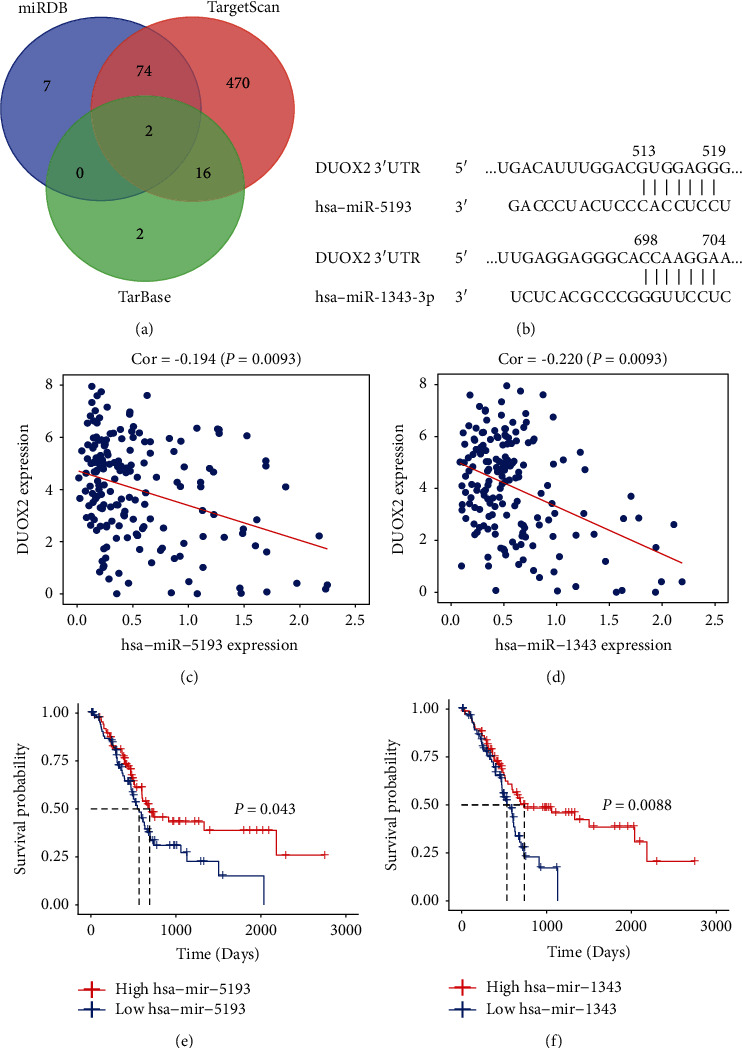
Prediction of miRNAs and DUOX2. (a) Potential regulatory miRNAs of DUOX2 present in 3 prediction and validation websites. (b) hsa-miR-5193 and hsa-miR-1343-3p binding putative sites of DUOX2 3′UTR. Correlation between (c) hsa-miR-5193, (d) hsa-miR-1343, and DUOX2 mRNA expression. Kaplan-Meier curves showing the expression of (e) hsa-miR-5193 and (f) hsa-miR-1343-3p related with overall survival in TCGA.

To predict the potential miRNA of DUOX2, three databases including the TargetScan, miRDB, and DIANA-TarBase were used. hsa-miR-5193 and hsa-miR-1343-3p putative binding sites in DUOX2 3′UTR were showed in Venn diagram (Figures 7(a) and 7(b)). Based on TCGA data, hsa-miR-5193 and hsa-miR-1343-3p showed weak and negative correlation with DUOX2 by liner regression analysis ((Cor = −0.194 and *P* = 0.009), (Cor = −0.220 and *P* = 0.003), respectively) (Figure 7(c)). K-M survival analysis indicated that high expression of hsa-miR-5193 and hsa-miR-1343 was correlated with favorable OS (*P* = 0.043 and *P* = 0.0088, respectively) (Figure 7(d)). These results demonstrated that elevated hsa-miR-5193 and hsa-miR-1343-3p may participate in downregulation of DUOX2 in PC.

## 4. Discussion

In the current study, DUOX2 could inhibit PC cell migration and invasion, as well as proliferation, implying that DUOX2 plays an important role in the development and progression of PC. Based on transcriptomics study, high expression of DUOX2 could be regarded as prognostic predictor for poor prognostic evaluation of patients with PC. These results suggest that DUOX2 can be used as a potential therapeutic target for advanced PC.

DUOX2 is associated with chronic inflammation including chronic inflammatory preneoplastic disorders, inflammatory bowel disease, and chronic pancreatitis [18]. Chronic inflammation plays an important role in carcinogenesis of certain types of cancers [9–11]. Recent oncological researches have revealed that DUOX2 is widely investigated and showed overexpression in many malignant tumors [18]. It has been reported that high DUOX2 levels contribute to the progression of PC in patients with chronic pancreatitis [13]. However, biological functions of DUOX2 in PC have not been elucidated till date. In our study, DUOX2 mRNA was shown to be dramatically upregulated in both PC tissues and cell lines when compared with matching adjacent normal tissues and cell lines, respectively. This result was in line with the bioinformatics data of TCGA and GTEx in PC by GEPIA. In addition, CCK-8 assay, transwell migration assay, and invasion assay were used to investigate the functional role of DUOX2. Knockdown of DUOX2 using siRNA technology revealed significant inhibition of cell proliferation, migration, and invasion of cancer cells *in vitro*. These results implied that DUOX2 might play an important role as a tumor promoter in PC. Moreover, how DUOX2 regulates PC is unclear, and the underlying molecular mechanisms require further elucidation.

For disease treatment, prediction of patient prognosis is vitally important because the treatment modality or therapeutic strategy varies based on patient prognosis. Therefore, novel biomarkers are necessary to enhance individual prognostication and precise therapies [26]. In our study, the results were compared between clinical and public GEPIA website based from TCGA and GTEx data. The main difference is that we obtained the clinical data from Asians, but vast majority of the GEPIA data is from Caucasians. Kaplan-Meier survival curve showed that low DUOX2 mRNA levels were significantly associated with favorable OS in PC patients, which was consistent with the public mRNA dataset obtained from TCGA and the GTEx databases. Above all, there was nearly no racial gap in the expression of DUOX2 and its effect on the OS of PC patients.

TNM stage, which is the major determinant of appropriate treatment and prognosis, records the primary and regional nodal extent of the tumor and the absence or presence of metastases [27]. Public data revealed that high DUOX2 expression was positively correlated with early TNM stage, while our clinical study analysis showed that it was positively associated with advanced TNM stage. That is because public data showed a large number of patients were in early stage (stages I and II), only sporadic patients were in late stage (stages III and IV), whereas all patients involved in our study were relatively even distributed among the TNM stages. Furthermore, our study disclosed that tumor diameter, T stage, and N stage were in relation to DUOX2 expression. However, no relationship was observed with other clinical features, such as gender, age, tumor location, and histologic grade. Univariate analysis revealed that DUOX2 expression, tumor location, TNM stage, and N classification were considered as prognostic indicators for OS. In addition, multivariate analysis suggested that high DUOX2 expression, head of pancreatic cancer, and advanced TNM stages were independent prognostic factors of poor OS. These results indicated that DUOX2 upregulation was highly correlated with PC development and progression. Of note, the association between the PC primary location site and survival is still a contradictory debate. Some recent study reported that pancreatic head tumor predicted better OS than body/tail of pancreatic cancer based on the multivariate Cox regression analysis regardless of whether the information was taken into account the same TNM classification [28–30]. In addition, previous studies were found that pancreatic primary tumor site has resembled OS rate, which is consistent or even inconsistent with multivariate Cox regression analysis [31–33]. However, a large population retrospective cohort study in the United States emphasized that pancreatic head tumor predicts worse OS compared to either body or tail of pancreatic cancer, which is aligned with our study result [34]. The reason for these controversial reports may due to the discrepancies of recruiting criteria for patients and TNM staging as well as biological characteristic between tumor sites.

So far, there is little research on the upstream regulatory mechanism of DUOX2; hence, our study initially explored the transcriptional regulatory mechanism from the perspective of bioinformatics. ONECUT2, ZBTB7B, NR1I2, ELF3, and TCF7L2 were moderately and positively associated with DUOX2, suggesting their low expression may result in low DUOX2 expression. It was reported that high TCF7L2 expression predicts worse OS in PC [35], and the expression of NR1I2 is not associated with OS in PC [36]. TCFL5, RAC3, CBX1, USF2, and RPA2 were weakly and negatively related to DUOX2, indicating that their high expression may lead to low expression of DUOX2. Moreover, a recent study revealed that N-alpha-acetyltransferase 10 (NAA10) is involved in the complex transcriptional regulation of PXR in PC cells [37]. Of these adverse regulatory TFs, only USF2 expression was significantly associated with OS in PC according to GEPIA. Strikingly, Song et al. demonstrated that RAC-3 acts as an oncogene and is negatively connected with OS in PDAC [38], which is contrary to our prediction result. In addition, there was no current study on ONECUT2, ZBTB7B, ELF3, TCFL5, CBX1, USF2, and RPA2 in PC; therefore, all of them need to be further investigated. Taken together, TCF7L2 may play a key role in regulating DUOX2 at the transcriptional level.

miRNAs are short RNA that partially binds target genes, thereby inhibiting their translation and regulating their expression and signaling pathways [39–42]. According to the three online databases, hsa-miR-5193 and hsa-miR-1343-3p have been verified negatively correlating with DUOX2 by linear regression analysis. Furthermore, both hsa-miR-5193 and hsa-miR-1343-3p are negatively associated with OS based on the PC date from TCGA, which need to be further confirmed by multivariate Cox regression analysis. There are sporadic reports about hsa-miR-5193 and hsa-miR-1343-3p. It has been reported that the role of hsa-miR-5193 inhibits the expression of TRIM11, resulting the better OS in prostate cancer, while it suppresses HBV replication [43, 44]. The latest study revealed that upregulation of hsa-miR-1343-3p results in CYP2C19 downregulation, and changes in CYP2C19 protein are negatively associated with the degree of clopidogrel therapy resistance because of its polymorphism in Indian population [45]. Stolzenburg et al. reported that hsa-miR-1343-3p plays a key role in preventing and treating diseases involving overactive TGF-*β* pathway and fibrosis [46, 47]. Apparently, the involvement of two miRNAs in the development of tumor is poorly investigated. This study has been predicted that the potential DUOX2 binding sites for hsa-miR-519 and hsa-miR-1343-3p; hence, it is reasonable to speculate that hsa-miR-519 and hsa-miR-1343-3p are negatively regulatory DUOX2, which need to be experimentally validated in vitro.

There are several limitations in our study that should be acknowledged. Firstly, studies at tissue level and in vitro only were carried out, lacking further experiments in vivo. Secondly, more clinical tissue samples and cell lines are needed to examine the biological functions of DUOX2. Thirdly, and most important of all, in order to avoiding selection bias, we should carry out a randomized and controlled trial.

In summary, our results indicated that DUOX2 plays a decisive role in PC progression and it could serve as a promising prognostic biomarker in PC. However, future increasing functional investigations, animal experiments, and verifying the above prediction of bioinformatics are warranted to further perform, thus exploring the underlying mechanisms of DUOX2 in PC occurrence.

## Figures and Tables

**Table 1:** Primer sequences for RT-qPCR and siRNA of DUOX2.

Gene		Primer sequences (5′-3′) or sequence of siRNA (5′-3′)
DUOX2	Forward	AATGGCCTCCCAGATTTCGG
	Reverse	CCCCATATCTCGGCCACGTTG
ACTB	Forward	TGACGTGGACATCCGCAAAG
	Reverse	TGACGTGGACATCCGCAAAG
DUOX2	si1406	GGAGGACAACAUAGUGGUUTT
	si2848	CCAUGAUGCGAUCCUUCAUTT

**Table 2:** Associate of DUOX2 mRNA expression with clinicopathological characteristics.

Variable	Group	Number of cases (%)	DUOX2 expression		*P* value
			High	Low	
Gender	Male	62 (72.1)	29	33	0.336
	Female	24 (27.9)	14	10	
Age (years)Age (years)	>65	37 (43.0)	17	20	0.514
	≤65	49 (57.0)	26	23	
Tumor diameter (cm)	>2	67 (77.9)	38	29	0.019^∗^
	≤2	19 (22.1)	5	14	
Tumor location	Head	57 (66.3)	27	30	0.494
	Body/tail	29 (33.7)	16	13	
TNM stage	I-IIA	43 (50)	15	28	0.005^∗∗^
	IIB-IV	43 (50)	28	15	
T stage	T1	20 (23.3)	6	14	0.041^∗^
	T2-3	66 (76.7)	37	29	
N stage	Absent	46 (53.5)	15	31	0.001^∗∗∗^
	Present	40 (46.5)	28	12	
M stage	M0	85 (98.8)	43	42	1
	M1	1 (1.2)	0	1	
Histological stage	I-II/II	31 (36)	15	16	0.822
	II-III/III	55 (64)	28	27	
Serum CA-199 (kU/L)Serum CA-199 (kU/L)	≥39	59 (72)	31	28	0.275
	<39	23 (28)	9	14	
Serum CEA (*μ*g/L)	≥4.7	29 (35.4)	13	16	0.596
	<4.7	53 (64.6)	27	26	
Perineural invasion	Absent	14 (16.5)	4	10	0.088
	Present	71 (83.5)	38	33	

^∗^ *P* < 0.05, ^∗∗^*P* < 0.01, and ^∗∗∗^*P* < 0.001.

## Data Availability

The data sets used and/or analyzed during the current study are available from the corresponding author on reasonable request.
